# Supplementary material for: Habits and perceptions regarding open science by researchers from Spanish institutions
Source: PLoS One. 2023 Jul 13;18(7):e0288313. doi: 10.1371/journal.pone.0288313 (PMC10343031; doi:10.1371/journal.pone.0288313)
Supplement: S1 File — (PDF) [file pone.0288313.s001.pdf]

1- Open Access

How many articles have you published in the past two years (2019-2020)?
Select one of the following options.
Please select **just one** of the following options:

- 1-3.
- 4-6.
- More than 6.

How many of these articles were published openly, whether in the journal itself or in an open-access institutional repository or collection?
Select one of the following options.
Please select **just one** of the following options:

- None.
- Fewer than half.
- Half or more.
- All.

By what means do you deposit published articles in journals?
Tick the corresponding options.
Please, tick the options that correspond:

- I deposit in an open-access repository the version of the published article when the licence permits it or I own the rights.
- I deposit in an open-access repository the accepted and reviewed version of the article when I have permission or own the rights.
- I deposit in an open-access repository the pre-print version of the article (submitted version not yet reviewed).
- I deposit on academic social networks (Researchgate, Academia.edu...) the version of the published article when the licence permits it or I own the rights.
- I deposit on academic social networks (Researchgate, Academia.edu...) the accepted and reviewed version of the article when I have permission or own the rights.
- I deposit on academic social networks (Researchgate, Academia.edu...) the pre-print version of the article (submitted version not yet reviewed).
- None (I do not deposit the published articles or pre-prints in a public-access repository or on academic social networks).

The deposited articles referred to research with funding that was…
Tick the corresponding options.
Please, tick the options that correspond:

- European or international
- National
- Autonomous community or local
- Private
- I do not currently have funding

The reasons for open publications are:
(select according to your level of agreement with the statement).
Please, assign a value from 1 to 5 to each concept, where 1 is “I strongly disagree” and 5 is “I strongly agree”.

- I believe that open access is a necessary condition of science.
- Open access enables one to comply with open-access policies of institutions or other entities.
- Open access increases the visibility of a publication.
- It is an exercise in transparency that serves as a way of being accountable to citizens so that society knows what is being done with public money.
- It is part of the logical exchange of knowledge with other colleagues.
- It is the only way in which colleagues in other countries can access my work.
- Open access provides publications with immediacy.
- Open access provides transparency in the publication’s business model.

- Open access promotes innovation in content and publication type (it attracts younger authors).
- Open access allows content to be reused and shared.
- Open access enables one to comply with open-access policies of institutions or other entities.

The reasons for NOT open publications are:
(select according to your level of agreement with the statement).
Please, assign a value from 1 to 5 to each concept, where 1 is “I strongly disagree” and 5 is “I strongly agree”.

- Normal publications in my discipline do not tend to be open access.
- Open-access journals tend to have less impact and this has repercussions for the assessment of research staff (six-year terms, etc.).
- Open-access publishing is not part of my research priorities.
- I do not have the economic support of my institution.
- I do not have the technical support of my institution.
- My lines of research do not align with the open sharing of content.
- I believe that open-access journals are low-quality.
- I do not want to risk open publishing in predatory journals.

Do any of your funding sources require open access to your publications?
Select one of the following options.
Please select **just one** of the following options:

- Yes.
- No.
- I don’t know.

By what means do you prefer to disseminate your open-access research work?
Select one of the following options.
Please select **just one** of the following options:

- Publishing in an open-access journal.
- Publishing with open access in a subscription journal (paying APCs, hybrid journals).
- Depositing a copy of the published article in an open-access institutional repository or collection.
- Depositing only the submitted version on a server for pre-prints.
- Other.

APCs (Article Processing Charges) are fees journal publishing houses sometimes charge authors of scientific works for their work to have open access, either because the journal has open access or because it is a hybrid journal (paid subscription journal and payment for publishing specific articles with open access).

What is your opinion of the following activities relating to research support by the Library in facilitating the publication of open-access articles?
(select according to the degree of usefulness you would assign each activity).
Please, assign a value from 1 to 5 to each concept, where 1 is “Not useful at all” and 5 is “Very useful”:

- Total or partial funding of the publication (payment of APCs).
- Support in choosing a journal.
- Training on open-access publishing.
- Support in aspects relating to intellectual property and editorial policy.
- Training in publishing in repositories and on social academic networks.

What is your opinion of the following activities relating to support by your institution in open-access publications?
(select according to the degree of usefulness you would assign each activity).
Please, assign a value from 1 to 5 to each concept, where 1 is “Not useful at all” and 5 is “Very useful”:

- Financing the costs of open publication
- Clear open-access policies.
- Technical support with publishing.

- Recognition and rewards for open publishing.

Do you have any comments about this section?
Please write your response here:

2- Publishing process

Have you needed to pay APCs to publish openly in recent years (2019-2020)?

Select one of the following options.
Please select **just one** of the following options.

- Yes.
- No.

In the process of choosing a journal, how relevant do you find the following criteria?
(select the relevance you give each criterion).
Please assign a value from 1 to 5 to each concept, where 1 is "Not relevant at all" and 5 is "Very relevant":

- It is and open access journal.
- Indexation in databases and quartiles.
- Discipline- based journal.
- Renowned publishing house.
- Time from submission to publication.
- Editorial policy relating to the depositing of articles in repositories.
- Authors keep copyright.
- That it does not require the payment of APCs.

APCs (Article Processing Charges) are fees journal publishing houses sometimes charge authors of scientific works for their work to have open access, either because the journal has open access or because it is a hybrid journal (paid subscription journal and payment for publishing specific articles with open access).

What is your opinion of APCs?
Tick the corresponding options.
Please tick the options that correspond.

- They are generally excessive.
- They are adequate.
- Sometimes they are excessive.
- They are necessary.
- Other:

Where did the budget for paying APCs come from?
Tick the corresponding options.
Please tick the options that correspond.

- Project funding.
- Research group funding.
- Aid from the university or the library.
- Other:

The reasons for paying APCs were:
(select according to your level of agreement with the statement).
Please, assign a value from 1 to 5 to each concept, where 1 is "I strongly disagree" and 5 is "I strongly agree".

- In my discipline it is rare to find open journals without APC payment.
- My financing entity obligates me to publish in open-access journals and in my discipline they are almost all paid.
- The publishing house and/or journal was chosen for the speed of its response.
- I think that APCs serve to guarantee the quality of scientific journals.
- The majority of hybrid journals in my discipline are indexed in recognized databases.

Do you have any comments about this section?
Please write your response here:

3- Open data

Does your funder have a specific policy for facilitating open research data?
Select one of the following options.
Please select **just one** of the following options:

- Yes.
- Yes, but my research type exempts me from the need to publish data openly (confidentiality, privacy...).
- No.
- I don't know.

Does your institution have specific policies for facilitating open research data?
Select one of the following options.
Please select **just one** of the following options:

- Yes.
- Yes, but my research type exempts me from the need to publish data openly (confidentiality, privacy...).
- No.
- I don't know.

Have you openly deposited your research data?
Select one of the following options.
Please select **just one** of the following options:

- Yes.
- No.
- No, but I will in the short- to medium-term future.

Where did you deposit it?
Only answer this question if the following conditions are met:
Your answer to question "20 [Q18]" (Have you openly deposited your research data?) was "Yes".
Tick the corresponding options.
Please tick the options that correspond.

- Institutional repository.
- Funder's repository.
- Discipline repository.
- Orphan repository (Zenodo, Figshare...).
- Research data repository (Dryad, Genbank, Dataverse...).
- Data journals.

What were your reasons for depositing it?
(select according to your level of agreement with the following statements).
Only answer this question if the following conditions are met:
Your answer to question "20 [Q18]" (Have you openly deposited your research data?) was "Yes".
Please assign a value from 1 to 5 to each concept, where 1 is "I strongly disagree" and 5 is "I strongly agree":

- Mandate by my funder
- Policy or mandate from my institution.
- To allow data to be reused.
- Exercise in transparency.
- Visibility of research results.
- Collaboration with other teams.
- Enables readers to access data files.
- Lends trustworthiness and reliability to the journal.
- Open data as complementary materials are an indicator of a journal's quality.
- Enables results to be validated.

What were your reasons for not depositing them?
(select according to your level of agreement with the following statements).
Only answer this question if the following conditions are met:
Your answer to question “20 [Q18]” (Have you openly deposited your research data?) was “No”.
Please assign a value from 1 to 5 to each concept, where 1 is “I strongly disagree” and 5 is “I strongly agree”:

- There was not a funder’s mandate
- Matters of privacy and confidentiality.
- Reluctance to share research data with the competene.
- Lack of knowledge of how to share research data.
- It is not possible to share my datasets .
- To avoid biased use or misuse of raw research data.
- It may compromise the future use of that data and potential economic interests.
- Open publishing of data can lead to its misuse.
- Open publishing of data can lead to its undue appropriation.
- I do not know where to deposit data so that it is locatable, accessible, interoperable and reusable.
- Open data as additional material in journals generates a degree of uncertainty as it has not yet been reviewed.

Do you believe the research data you deposited will be used?
Only answer this question if the following conditions are met:
Your answer to question “20 [Q18]” (Have you openly deposited your research data?) was “Yes”.
Select one of the following options.
Please select **just one** of the following options:

- Yes.
- No.

Do you often use research data shared by your colleagues?
Select one of the following options.
Please select **just one** of the following options:

- Yes.
- No.

What are your reasons for not using it?
(select according to your level of agreement with the following statements).
Only answer this question if the following conditions are met:
Your answer to question “25 [Q23]” (Do you often use research data shared by your colleagues?) was “No”.
Please assign a value from 1 to 5 to each concept, where 1 is “I strongly disagree” and 5 is “I strongly agree”:

- Lack of technical knowledge of data handling.
- Mistrust in open raw data.
- I prefer to work with accepted results published in articles.
- Ethical reasons.

Do you think your institution should provide you with more support in depositing research data?
Select one of the following options.
Please select **just one** of the following options:

- Yes.
- No.

What type of support would you like your university or library to offer you with regard to open research data?
Tick the corresponding options.
Please tick the options that correspond.

- Advice in open data management.

- Training in open data management.
- Support with writing data management plans.
- Support with managing metadata, interoperability standards, DOIs...
- Support with ethics and the anonymization of data.
- Servers for sharing research data.
- Other:

Do you have any comments about this section?
Please write your response here:

4- Open peer review

Do any of the journals where you have published in the last two years (2019-2020) have an open peer review system?
Select one of the following options.
Please select just one of the following options:

- Yes.
- No.

Open Peer Review refers to one of the following peer review modalities:
1. Open identities: both the reviewer and the author know each other's identity.
2. Open reports: review reports are published along with the research article.
3. Open participation: the general community can contribute to the review process.

In the last two years, have you reviewed an article for a journal with an open review system?
Select one of the following options.
Please select **just one** of the following options:

- Yes.
- No.

Did you agree for your review to be made public (if this was optional)?
Only answer this question if the following conditions are met:
Your answer to question "31 [Q28]" (In the last two years, have you reviewed an article for a journal with an open review system?) was "Yes".
Select one of the following options.
Please select **just one** of the following options:

- Yes.
- No.

What is your opinion of Open Peer Review?
(select according to your level of agreement with the following statements).
Please assign a value from 1 to 5 to each concept, where 1 is "I strongly disagree" and 5 is "I strongly agree":

- It is alright, but not in my discipline.
- It can help improve the form and depth of reviews.
- Anonymity prevents fear of one's identity being discovered, particularly among younger researchers.
- The more transparency, the better.
- It can generate conflicts of interest between the parties (reviewer-reviewed, young researcher-senior, geographical bias...).
- It can benefit and foster scientific debate.

Do you have any comments about this section?
Please write your response here:

5- COVID-19 Effect

In your view, has the ongoing pandemic had an effect on your rate of article publication?
Select one of the following options.

Please select just one of the following options.

- Yes, I have published more.
- Yes, I have published less due to the crisis and its health and social effects.
- Yes, I have published less due to overload of teaching work (move to virtual...).
- It has had no significant effect on my rate of publication.

Do you have any comments about this section?
Please write your response here:

6- Final considerations

Do you know what Open Science means?
Select one of the following options.
Please select **just one** of the following options.

- Yes, and I agree with its postulates and proposals.
- Yes, but I think the transition needs to occur more slowly.
- Yes, but I think it involves a change for which the academic system is not yet ready.
- I have heard of it, but I don't know what it's about.
- I have never heard of it.

Would you agree with a system by which all articles were open and it was only necessary to pay to publish?
Select one of the following options.
Please select just one of the following options.

- Yes.
- No.

Do you think that scientific journals as they exist today will still be around 15 years from now?
Select one of the following options.
Please select **just one** of the following options.

- Yes, I hope so.
- Yes, unfortunately.
- No, I hope not.
- No, unfortunately.

Do you think that in the future all journal articles will be open?
Select one of the following options.
Please select just one of the following options.

- Yes.
- No.

Do you know the principles and objectives of Plan S (https://www.coalition-s.org/why-plan-s/) for accelerating the transition towards open access?
Select one of the following options.
Please select **just one** of the following options.

- Yes, and I agree with them.
- Yes, and I don't agree with them/.
- No, I don't know them.

Do you have any comments about this section?
Please write your response here:

7- Sociodemographic questions

Gender
Select one of the following options.
Please select just one of the following options.

- Female.

- Male.
- Non-binary.
- Gender fluid.
- Prefer not to say.

Age
Select one of the following options.
Please select **just one** of the following options.
- 21-35.
- 36-50.
- 51-65.
- Over 65.

Type of centre where you carry out your normal working activity
Tick the corresponding options.
Please tick the options that correspond:
- Public university.
- Private university.
- Public research centre.
- Private research centre.
- Other:

Discipline
Select one of the following options.
Please select **just one** of the following options.
- Social Sciences.
- Arts and Humanities.
- Health Sciences.
- Engineering.
- Life sciences.
- Experimental sciences.
- Maths and physics.

Thanks for your participation from the research team *Acceso Abierto a la Ciencia* (https://www.opensciencespain.org).

Ernest Abadal, IP

CIENCIA ABIERTA EN ESPAÑA: UNA APROXIMACIÓN GLOBAL PARA EVALUAR SU GRADO DE IMPLEMENTACIÓN

OPEN SCIENCE IN SPAIN: A GLOBAL APPROACH TO ASSESS ITS DEGREE OF IMPLEMENTATION

RTI2018-094360-B-I00

Submit your survey
Thank you for completing this survey.
