# Supplementary material for: Habits and perceptions regarding open science by researchers from Spanish institutions
Source: PLoS One. 2023 Jul 13;18(7):e0288313. doi: 10.1371/journal.pone.0288313 (PMC10343031; doi:10.1371/journal.pone.0288313)
Supplement: Attachment — Submitted filename: Response to Reviewers.docx (1).pdf [file pone.0288313.s002.pdf]

Response to Reviewers

PONE-D-23-01585

Habits and perceptions regarding open science by researchers from Spanish Institutions

Reviewer's Responses to Questions

Comments to the Author

1. Is the manuscript technically sound, and do the data support the conclusions?

The manuscript must describe a technically sound piece of scientific research with data that supports the conclusions. Experiments must have been conducted rigorously, with appropriate controls, replication, and sample sizes. The conclusions must be drawn appropriately based on the data presented.

Reviewer #1: Partly

Reviewer #2: Yes

Reviewer #3: Yes

---

2. Has the statistical analysis been performed appropriately and rigorously?

Reviewer #1: No

Reviewer #2: Yes

Reviewer #3: Yes

---

3. Have the authors made all data underlying the findings in their manuscript fully available?

The PLOS Data policy requires authors to make all data underlying the findings described in their manuscript fully available without restriction, with rare exception (please refer to the Data Availability Statement in the manuscript PDF file). The data should be provided as part of the manuscript or its supporting information, or deposited to a public repository. For example, in addition to summary statistics, the data points behind means, medians and variance measures should be available. If there are restrictions on publicly sharing data—e.g. participant privacy or use of data from a third party—those must be specified.

Reviewer #1: Yes

Reviewer #2: No

Reviewer #3: No

4. Is the manuscript presented in an intelligible fashion and written in standard English?

PLOS ONE does not copyedit accepted manuscripts, so the language in submitted articles must be clear, correct, and unambiguous. Any typographical or grammatical errors should be corrected at revision, so please note any specific errors here.

Reviewer #1: Yes

Reviewer #2: Yes

Reviewer #3: Yes

---

5. Review Comments to the Author

Please use the space provided to explain your answers to the questions above. You may also include additional comments for the author, including concerns about dual publication, research ethics, or publication ethics. (Please upload your review as an attachment if it exceeds 20,000 characters)

Reviewer #1: Habits and perceptions regarding open science by researchers from Spanish Institutions. Manuscript No.: PONE-D-23-01585

Thank you for the time you have spent reviewing the article and for your comments and suggestions. We are going to respond in an orderly manner below to each reviewer and each topic. We will also add them in the corresponding places within the manuscript.

This is a paper that describes the results of the online survey on open science in Spain indicated different perceptions of researchers. However, in its current version, I cannot support publishing this paper in the journal Plos One. The reasons are the following:

1. The abstract should include an underscore the scientific value added of paper, this section, it must structure in only one paragraph and include the main conclusions and implications.

In terms of the first question, we have added, in a summarized format, the added value and main conclusions as well as changing the structure of the summary to a single paragraph.

"This research offers an in-depth picture of researchers, as one of the main actors to whom this transformation and implementation of open science will fall. It focuses on the different aspects of OS: open access, open data, publication process and open review in order to identify habits and perceptions. This is to make possible an implementation of the movement".

We have also added to the methodology section a mention of its limitations as a qualitative study.

"Despite the limitations regarding the representativeness of the results of a qualitative study, as well as the WOSI for obtaining contact emails, the research makes it possible to identify the habits and perceptions of Spanish researchers".

2. In the introduction, the authors show a background and context of study. However, this section could explain in more detail: What is the new knowledge or value added of study? What is the research question or objectives? What makes the applied methodology suitable and superior in comparison to existing studies? What is the expected new insight gained by applying the method?, this section could include structure of manuscript and to improve literature review.

We have incorporated the questionnaire as an appendix and included the added value which was not explicit, as well as objectives, research questions and specification of what the methodology used contributed. We have also incorporated the structure of the article in order to situate the reader and some of the recommended bibliographical references.

"The following research questions (RQ) will be addressed:

RQ1: What are the perceptions of researchers regarding open access, open data, publication process and open peer review in the Spanish scholarly publishing landscape?

RQ2: What insights does this give us into research practices related to open science?”

3. In the second section, authors describe methodology. This section needs to improve description of instrument with questions per category and the theoretical support, sample and strategies to analysis that guarantee robustness and reliability of the results.

A technical description of the sample has been incorporated, as well as the parts of the questionnaire.

“A script scanned the bibliographic references file line by line to find those beginning with “CORRESPONDENCE ADDRESS:” and containing ‘Spain’ and extracted from them a word pattern compatible with an email address.”

The questionnaire consisted of five parts:

1. Demographic data about the researcher and the profile of the respondent
2. Information in terms of open access;
3. Information in terms of the open peer review process;
4. Information related to the publication process;
5. Information related to the underlying data of published papers.
6. Information related to COVID.

Depending on the question, responses could be single, multiple or 5-point Likert scale responses (e.g., from “strongly disagree” to “strongly agree”), which were translated into a numerical scale from 1 to 5 for quantitative analysis and statistical processing.

4. Third section describes the main results; this section is adequate. However, the authors should explain significance level and statistics tests used.

4. Discussion must be improved including an analysis of results in comparison with literature strengths and shortcomings of this analysis and a short outlook on further research requirements and possible research extensions.

The bibliography has been extended in order to enrich the results. Some of the references provided include:

Aleixandre-Benavent R, Vidal-Infer A, Alonso-Arroyo A, Peset F, Ferrer Sapena A. “Research Data Sharing in Spain: Exploring Determinants, Practices, and Perceptions” Data 5; 2020, no. 2: 29. https://doi.org/10.3390/data5020029

De Filippo D, Mañana-Rodriguez J. The practical implementation of open-access policies and mandates in Spanish public universities. Scientometrics, 2022, 127, 7147–7167. https://doi.org/10.1007/s11192-021-04261-x

Ruiz-Pérez S, Delgado-López-Cózar E. Spanish researchers’ opinions, attitudes and practices towards open access publishing. El profesional de la información, 2017, v. 26, n. 4, pp. 722-734. https://doi.org/10.3145/epi.2017.jul.16

We hope to have more productivity in the medium term from the current project "Open Science in Spain: the transition to a new research model" (PID2021-125828OB-I00) a new edition of what we present in this paper.

5. Conclusions are adequate.

I wish that these comments could help the author to improve the paper.

——

Reviewer #2: The manuscript (text, tables and figures), "Habits and perceptions regarding open science by researchers from Spanish Institutions", focuses on the situation of open science in Spain and the results of a survey of Spanish researchers in 2021.

Major revisions

The bibliographic review refers both to declarations (senso lato), standards and laws (UNESCO, BOAI, European Union, Spanish rules...) as well as to scientific articles. In both aspects it is suggested to deepen the search for works on the habits of researchers, in order to offer a broader discussion. I also suggest insisting on reviewing in the previous literature other relevant topics, as these: how erratic the forms of results dissemination continue to be; the extreme situation of this phenomenon in the case of research data given their intrinsic diversity, in comparison with open access… This makes the difference between the success of the openning the articles and the difficulties for the research data.

We have taken your observations into consideration and incorporated some more bibliographical references in order to offer a broader discussion.

Some of the references include:

Aleixandre-Benavent R, Vidal-Infer A, Alonso-Arroyo A, Peset F, Ferrer Sapena A. "Research Data Sharing in Spain: Exploring Determinants, Practices, and Perceptions" Data 5; 2020, no. 2: 29. https://doi.org/10.3390/data5020029

De Filippo D, Mañana-Rodriguez J. The practical implementation of open-access policies and mandates in Spanish public universities. Scientometrics, 2022, 127, 7147–7167. https://doi.org/10.1007/s11192-021-04261-x

Ruiz-Pérez S, Delgado-López-Cózar E. Spanish researchers' opinions, attitudes and practices towards open access publishing. El profesional de la información, 2017, v. 26, n. 4, pp. 722-734. https://doi.org/10.3145/epi.2017.jul.16

The methodology used is rigorous for the field being analysed, including a more sophisticated multi-factor analysis for the Likert scale questions. I only have one question: are all the questions asked in the questionnaire reflected in the results or are some not included in the study? If so, please explain why.

Not all the questionnaire responses were analysed, but rather a selection. We make use of the question to state that a final report of the project has been produced (Abadal, E. *et al*,. 2023) which compiles the questions not in the article, essentially for reasons of interest,

selection, length and limits. The report also compiles the work undertaken with the other 4 actors: vice rectors, quality agencies, librarians and journal editors (cited in the body of the article).

The results are perfectly described, although it is worth noting that, in the figures, the questions are cut off. If it is not possible to show them in full, it is suggested that the questionnaire was published. If they are in the text itself when the data are given, it is suggested that they be highlighted with underlining/bold/quotes or any other typographical means.

We have translated the questionnaire in order for it to be published and it will form part of the article in the appendix (OpenScienceSpain - Questions).

The Discussion and Conclusions section is a bit sparse. As I have said, it is imperative to expand the literature review in order to this section went beyond the survey results presented. A comparison of the results in Spain with the situation in other countries would enrich the conclusions offered by the work. One major issue that emerges from the survey responses, the evolution of open access towards the APC payment model, is treated rather lightly. Being a global concern that affects countries with less investment in research, such as Spain, and which is expected to last over time, it is necessary to work on the bibliography in greater depth, for which http://hdl.handle.net/1866/21676 or https://doi.org/10.5281/zenodo.4558704 are suggested. Other results concerning research data can be found at https://doi.org/10.1371/journal.pone.0021101 or even for Spain at https://doi.org/10.3390/data5020029.

This comment is shared by the other two reviewers, so we have worked in depth on the bibliographical review, providing some new works as well as considering them for discussion.

Minor revisions: Thank you for detecting the errors, they have been corrected directly in the appropriate places within the manuscript.

-- “Open Access data Polis funder and institution” in this sentence, I think should be policies the term polis
-- “In terms of researchers’ opinions on open peer review (Figure 14)” I think it’s figure 13
-- “article 12 in the “Fostering of open science and Civic Science”“ I think it is more widely translated as citizen science.

*3. Have the authors made all data underlying the findings in their manuscript fully available?
Not mentioned

The data has been deposited in CORA. https://www.csuc.cat/es/servicios/cora-repositorio-de-datos-de-investigacion

Linked reference: Ollé, C. *et al.*, (2023). Dataset on habits and perceptions of open science of researchers at Spanish institutions https://doi.org/10.34810/data690.

This is a private link until the article is published: https://dataverse.csuc.cat/privateurl.xhtml?token=ca21adc8-de69-43d9-8358-47448d92b6e4

Reviewer #3: There are some issues with the plots. Some of the legends are not translated from Spanish. Many of the questions are trimmed, e.g.: Most hybrid journals in my discipline a... This is likely to be solved at editing stage. I find slightly confusing the fact that "Yes" is represented in orange (red?) and "No" in green in some of the questions. Again, maybe for the editor to decide.

Thank you for the observations and considerations, we are sorry that the figures have had errors detected when uploaded to the platform as images. They have been corrected and their colours changed for the purposes of more logical interpretation and reading. For this second round of revision, we have also adapted the figures to journal guidelines.

It would be nice to compare these results with other countries with wider adoption of Plan S. Does it have any sort of influence in author's perceptions of OA? Maybe for a follow-up article.

The focus of the research as well as the project was centred around Spain, but it would be very interesting to establish a comparison at a European level, for example. For the current project [https://opensciencespain.org/presentacio/el-projecte/ PID2021-125828OB-I0] we are analysing the progress of other countries in order to identify good practice and models and suggest them for Spain.

I couldn't find table 4 ("Table 4 shows the number of factors obtained for each of the blocks of questions together with the KMO value which indicates the robustness of the factorial analysis"). It would be important to see it in order to validate the factorial analysis. Corrected.

---

6. PLOS authors have the option to publish the peer review history of their article (what does this mean?). If published, this will include your full peer review and any attached files.

If you choose "no", your identity will remain anonymous but your review may still be made public.

Do you want your identity to be public for this peer review? For information about this choice, including consent withdrawal, please see our Privacy Policy.

Reviewer #1: No

Reviewer #2: No

Reviewer #3: **Yes:** Sergio Ruiz-Perez
